# The Optimal Application of Medium Potency Topical Corticosteroids in Preventing Laser-Induced Inflammatory Responses—An Animal Study

**DOI:** 10.3390/life11040350

**Published:** 2021-04-17

**Authors:** Kuang-Ling Ou, Chia-Cheng Wen, Ching-Ya Lan, Yu-An Chen, Chih-Hsin Wang, Yi-Wen Wang

**Affiliations:** 1 Division of Plastic and Reconstructive Surgery, Department of Surgery, Tri-Service General Hospital, National Defense Medical Center, Taipei 114, Taiwan; kuanglio@usc.edu; 2 Division of Colon and Rectal Surgery, Department of Surgery, Tri-Service General Hospital, National Defense Medical Center, Taipei 114, Taiwan; wjason@mail2000.com.tw; 3 Department and Graduate Institute of Biology and Anatomy, National Defense Medical Center, Taipei 114, Taiwan; angie.lan@yuva.com.tw; 4 Department of Health and Leisure Management, Yuanpei University of Medical Technology, Hsinchu 300, Taiwan; yuanchen@mail.ypu.edu.tw

**Keywords:** laser therapy, wound healing, corticosteroids, acute inflammatory responses, scar contracture

## Abstract

Background: During ablative fractional resurfacing (AFR) laser therapy, thermal damage to the skin is inevitable, resulting in inflammatory responses and small wounds. Corticosteroids are known for their anti-inflammatory effect. However, inappropriate application of corticosteroids carries the risk of delayed wound healing. Therefore, we aimed to find the optimal administration route, timing, and duration of medium potency corticosteroid treatment to prevent AFR laser-induced inflammatory responses and to minimize the risk of delayed wound healing. Methods: We determined the anti-inflammatory efficacy of corticosteroids by skin erythema and tissue biopsies on C57BL/6 mice. Wound healing was evaluated by crust area and epithelial gap. Finally, Masson’s trichrome stain and α-SMA immunohistochemistry stain were used to analyze scar contracture. Results: Our results demonstrated that one dose of medium-potency topical corticosteroid applied immediately after AFR laser treatment could prevent erythema effectively with minimal disruption to wound healing. Notably, when more than one dose was administered, wound healing was delayed and scar contracture was aggravated by the application of medium-potency topical corticosteroids in a dosage-dependent manner. Conclusion: Our findings suggested that single-dose medium-potency topical corticosteroids could potentially improve AFR laser-induced acute inflammatory responses in clinical applications.

## 1. Introduction

The ablative fractional resurfacing (AFR) laser has been widely used clinically to treat acne, scars, photoaging, rhytides, laxity, morphea, and for drug delivery [1]. AFR lasers produce hundreds of microscopic thermal zones (MTZs) within a small area. MTZs heal fast because the nearby undamaged tissue facilitates rapid wound re-epithelization [2,3,4] and induces dermal remodeling of the lesion site by promoting the collagen synthese and lysis cycle. As a result, MTZs seldom lead to scar formation [5,6]. Adverse post-treatment responses to AFR laser therapy include erythema, swelling, blistering, oozing, crusting, delayed wound healing, post-inflammatory hyperpigmentation (PIH), and scarring [7,8]. These side effects are usually not detrimental or permanent; however, they are upsetting and lead to considerable psychological stress.

These unfavorable post-treatment responses are associated with acute inflammatory responses to the cutaneous thermal damage [2,9,10]. To reduce post-laser inflammatory responses, anti-inflammatory treatments such as ice packing [5] or administration of nonsteroidal anti-inflammatory ingredients [9,10,11] are employed. Among those strategies, corticosteroids have been shown to be more efficient than other measures [12] at reducing skin inflammatory responses such as UVB-induced erythema and melanin index [13], radio therapy-induced acute dermatitis [14], and laser treatment-induced erythema, swelling, and PIH [5,10,15]. Therefore, it is reasonable to assume corticosteroids may improve the inflammatory side effects of AFR laser treatment.

However, the application of corticosteroids can lead to delayed wound healing and potentially put patients at risk. In addition to the anti-proliferative effect of corticosteroids on fibroblasts, they also compromise DNA synthesis and mitosis resulting in decreased ECM production [16,17]. On the other hand, wound inflammation delays healing and causes scar formation. The wounded surface area after AFR laser treatment is about 5% to 20% of the total treatment surface area, on average. Thus, it is crucial to evaluate the advantages and disadvantages of applying corticosteroids in the prevention AFR laser-induced inflammatory responses.

Based on their strength, corticosteroids are categorized as low, medium, high, and super-high potency. While low to medium potency corticosteroids are recommended for acute inflammatory skin lesions according to guidelines [18], their anti-inflammatory effects are weak which limits their potential in reducing inflammatory responses to AFR laser treatment. While high or super-high potency steroids improve erythema after laser treatment, the risks of acneiform eruption and delayed wound healing increase [15,19]. In this study, we aimed to find the optimal administration route, timing, and duration for medium potency corticosteroid application to prevent AFR laser-induced inflammatory responses and to minimize the risk of delayed wound healing.

## 2. Materials and Methods

### 2.1. Animals

C57BL/6 mice were purchased from the BioLASCO (New Taipei City, Taiwan). All procedures were approved by the Institutional Animal Care and Use Committee of the National Defense Medical Center (IACUC-16-353). Six-week-old C57BL/6 mice were sedated with an intraperitoneal injection of Zoletil^®^ (20 mg/kg) (Virbac, Carros, France) plus Rompun^®^ (6 mg/kg) (Bayer, Gyeonggi-do, Korea). Mice were then shaved using a hair clipper (Oster, Hilliard, OH, USA) and depilated using hair removal cream before laser treatment. Mice were treated with or without betamethasone and euthanized at day 9 or day 21 post laser treatment. Wounds were harvested, formalin fixed, and embedded in wax for paraffin sectioning.

### 2.2. Corticosteroid Administration

Betamethasone is one of the medium potency corticosteroids, and widely used in clinical to improve inflammatory symptoms. In this study, betamethasone cream 0.06% (Rinderon-V^®^, Sinphar, Yilan City, Taiwan) served as the topical agent for betamethasone valerate, and 10 mg was applied to each wound in the topical corticosteroid group. Betamethasone 0.5 mg/kg (Rinderon, China Chemical & Pharmaceutical Co, Hsinchu, Taiwan) served as the subcutaneous injection (SC) agent for corticosteroid. The SC dosage was determined by pilot study, and this dosage was in the moderate dose range of corticosteroid pharmacological therapy.

### 2.3. Laser Treatment

Fractional 10,600-nm carbon dioxide laser (UltraPulse SurgiTouch CO_2_ Laser, Lumenis Ltd., Yokneam, Israel) treatment was performed by a single physician, using the same treatment parameters. Two 1 × 1 cm square laser lesions were made on the dorsal skin of each mouse, using pulse energy of 50 mJ. The spot size was 0.12 mm and static operating mode was used to achieve a 5% coverage area.

### 2.4. Skin Color Measurement

After laser treatment, the color of the lesion site was measured using a color reader (CR-10, Konica Minolta, Osaka, Japan). This color reader is a handheld contact measuring device that records color measurements in CIE-L*a*b* values. The CIE-L*a*b* color space system was defined by the International Commission on Illumination (CIE) in 1976. The L* value correlates to perceived color brightness from black (0) to white (100); a* from green (−) to red (+), and b* from blue (−) to yellow (+).

### 2.5. Wound Area Analysis

Images of laser lesion sites were collected at 30, 60, 120 min post treatment, and on days 7, 9, 12, 14, 21 post treatment. Photographs were acquired in a standardized method using a digital camera (D3100, Nikon, Tokyo, Japan) under identical camera settings, lighting, and distance, and were stored as JPG files. Wound images at day 9 were traced to determine crust area using image analysis software (Toupview, ToupTek Photonics Hangzhou, China). Skin specimens of lesion sites were collected at 2 h after laser treatment and on day 9, and treated with hematoxylin and eosin (H&E) stain to elucidate wound swelling and epithelization. The epithelial gap is the distance between the right and left tips of the epithelial tongues and was measured using Toupview software (Toupview).

### 2.6. Masson’s Trichrome Stain and Immunohistochemistry

Skin specimens from the lesion sites were collected at day 21, fixed with 10% formalin, embedded in paraffin, and cut into 6 μm paraffin sections. These were deparaffinized and rehydrated prior to staining. A trichrome stain kit (ScyTek Laboratories, Logan, UT, USA) containing Masson’s trichrome stain was used to observe the collagen fiber deposition. Immunohistochemistry was performed via the ABC method using a Vectastain ABC HRP kit (Vector Laboratories, Burlingame, CA, USA). A citrate-based antigen unmasking solution (Vector Laboratories) was used for antigen retrieval, and an alpha smooth muscle actin (α-SMA) antibody (GTX629702, GeneTex, Hsinchu, Taiwan) was used to target myofibroblasts. Sections were counterstained with Mayer’s hemalum solution (Merck Millipore, Darmstadt, Germany).

### 2.7. Statistical Analysis

Data analysis was conducted using Microsoft Excel (Microsoft, Redmond, WC, USA, version 16.43) and GraphPad Prism (GraphPad Software, San Diego, CA, USA, version 5.0). Intergroup comparison of L*a*b* data was compared by the Student’s t-test. Crust areas and epithelial gaps at different dosages were analyzed with one-way analysis of variance (ANOVA).

## 3. Results

### 3.1. The Administration Route Effects of Corticosteroids on the Improvement of Post-Laser Inflammatory Responses

**Figure 1:**
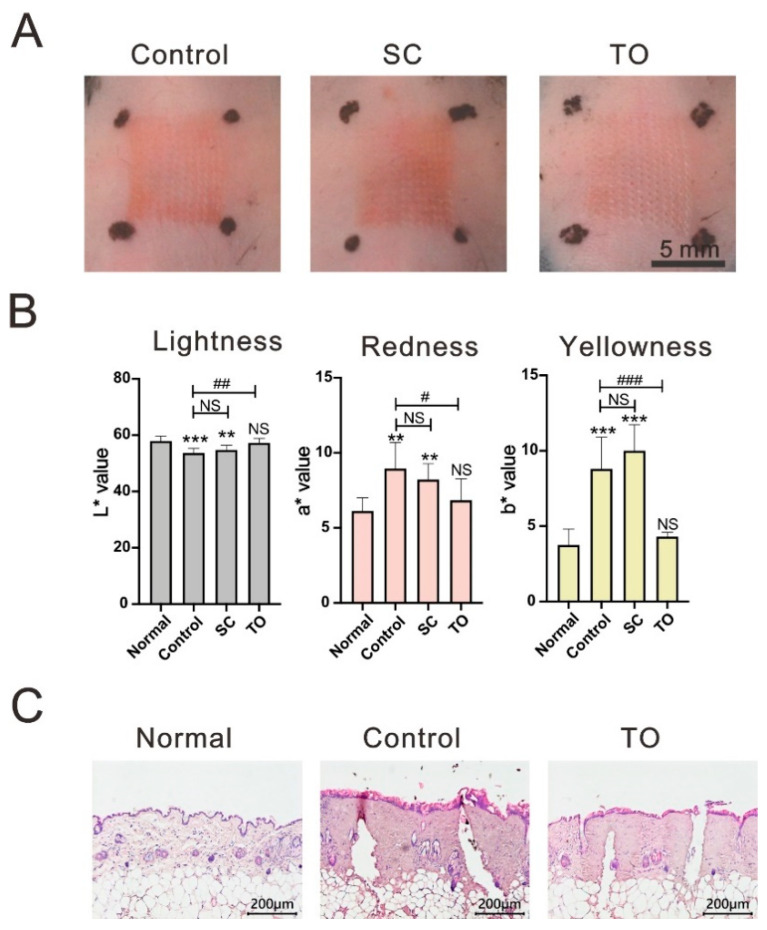
The administration route effects of corticosteroids on the improvement of post-laser inflammatory responses. (**A**–**C**) The dorsal skin 2 h following CO_2_ laser treatment. SC: subcutaneous injection 0.5 mg/kg betamethasone; TO: topical application 0.06% betamethasone. (**A**) Representative gross pictures of dorsal skin lesion sites. (**B**) The quantitative skin color (L*a*b* values), data represent mean ± S. D., *n* = 6; *: *p* < 0.05, **: *p* < 0.01, ***: *p* < 0.001 compared with normal; #: *p* < 0.05, ##: *p* < 0.01, ###: *p* < 0.001 compared with control, NS: Not significant. (**C**) Microscopic image of normal dorsal skin and laser lesion sites w/wo topical corticosteroids.

To optimize the administration route of corticosteroids in the treatment of skin inflammatory responses, we compared two different administration routes: subcutaneous injection 0.5 mg/kg of betamethasone solution (SC group) and topical application betamethasone cream (TO group). Total 20 mice were used for figuring out the administration route effects, 7 for control, 6 for SC, and 7 for TO. Subcutaneous corticosteroid injections were administered 30 min before laser treatment and topical corticosteroid applications were administered immediately after laser treatment. Skin redness was visible in the CO_2_ laser-lesioned control and SC groups, but was not obvious in the TO group at 120 min following CO_2_ laser treatment (Figure 1A). We used the CIE-L*a*b* color space system to quantize relative color of laser treated skin. The L* values of lesion sites were significantly higher in the TO group than in the control group (*p* < 0.01), but there was no significant color difference between the TO group and normal skin. The a* and b* values of lesion sites were significantly higher in the control and SC groups than in normal skin, but no difference was observed in these values between the TO group and normal skin (Figure 1B). Based on these findings, topical application of corticosteroids appears to be better for improving post-laser inflammatory responses than subcutaneous injection. Microscopic images of dorsal skin lesion sites showed that the skin tissue continued swelling at 120 min after CO_2_ laser treatment and that topical corticosteroids could partially improve the swelling (Figure 1C). This suggests that topical corticosteroids could decrease post-laser inflammatory responses.

### 3.2. The Administration Order Effects of Topical Application of Corticosteroids on Post-Laser Skin Erythema

**Figure 2:**
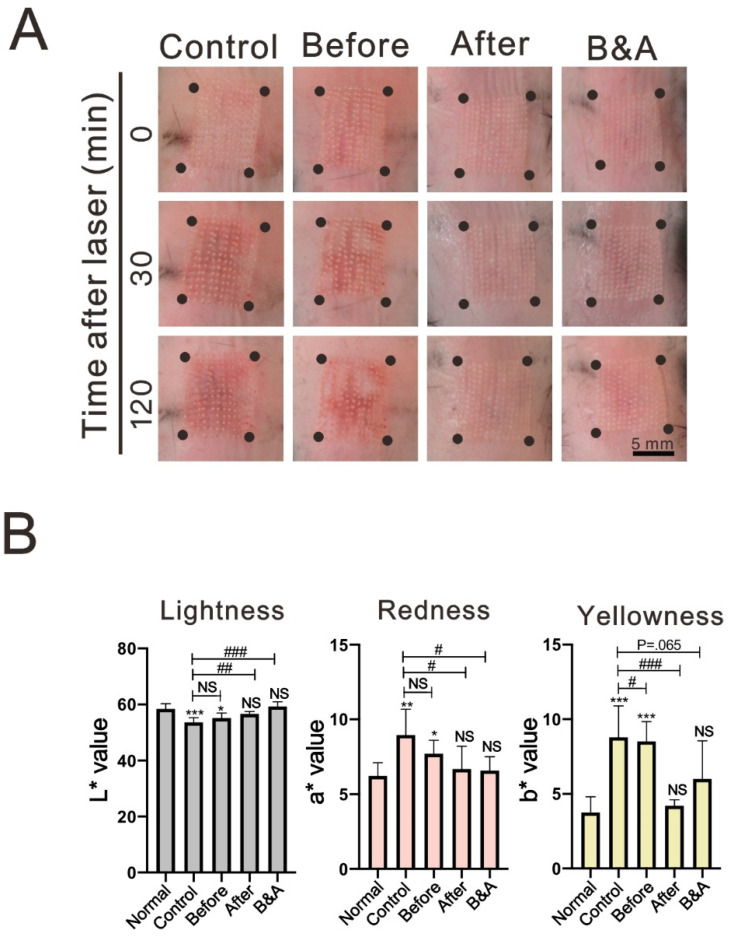
The order effects of topical corticosteroid treatment on the laser treated skin. (**A**) Representative pictures of dorsal skin lesion sites from mice from the control group and topical corticosteroid applied before, after, or B&A (before and after) with CO_2_ laser treatment, at 0, 30, and 120 min after CO_2_ laser treatment. Scale bar = 5 mm. (**B**) LAB values of dorsal skin lesion sites were analyzed at 120 min after laser treatment. Data represent mean ± S. D., *n* = 6. *: *p* < 0.05, **: *p* < 0.01, ***: *p* < 0.001 compared with normal skin; #: *p* < 0.05, ##: *p* < 0.01, ###: *p* < 0.001 compared with control, NS: Not significant.

To determine the administration order effects of corticosteroids on CO_2_ laser-induced skin redness, we applied topical betamethasone before, after, or before and after (B&A) CO_2_ laser treatment. The mice were divided into control (no betamethasone), before, after, and B&A groups. Total 28 mice were used for determining the administration order effects, 7 mice for each group. At 30 min after CO_2_ laser treatment, redness was prominent in the control and before groups (Figure 2A). However, there was no obvious redness in the after and B&A groups. At 120 min after CO_2_ laser treatment, redness was still evident in the control and before groups, whereas only slight redness was observed in the after and B&A groups. When compared with normal skin, the L*a*b* values of lesion sites at 120 min after CO_2_ laser treatment were significantly different in the control and before groups, but not in the after and B&A groups (Figure 2B). Our results indicate that applying topical corticosteroids immediately after CO_2_ laser treatment could sufficiently improve skin erythema.

### 3.3. Dosage-Dependent Effect of Topical Corticosteroids Delayed Cutaneous Wound Healing

**Figure 3:**
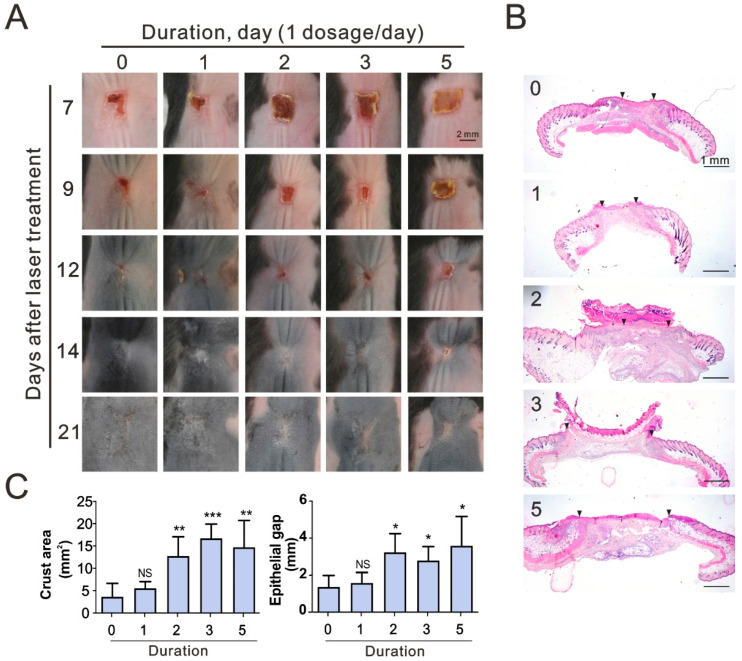
The dosage-dependent effects of topical corticosteroids on the cutaneous wound healing of mice. (**A**) Representative pictures of dorsal skin lesion sites with different administration durations at days 7, 9, 12, 14 and 21 after laser treatment. Scale bar = 1 mm. (**B**) Representative photomicrographs of repaired tissue at day 9 with H&E stain. The inverted triangles mark the tips of the epithelial tongues. Scale bar = 1 mm. (**C**) Crust area and epithelial gap at day 9, data represent mean ± S. D. *: *p* < 0.05, **: *p* < 0.01, ***: *p* < 0.001, NS: Not significant.

Studies indicate that corticosteroids can decrease inflammatory responses; however, they are also known to hamper wound healing [20]. Betamethasone has been shown to inhibit the proliferation of keratocytes and the production of collagen in corneal wounds [21]. Therefore, it is important to find a suitable administration period over which to reduce the laser-induced inflammatory response while not interfering with wound healing. The mice were divided 5 groups and a single dose of betamethasone was applied to the CO_2_ laser lesion sites daily over a period of zero (control), one, two, three, and five days. Total 37 mice were used for analyzed the dosage-dependent effect of topical corticosteroids, 8 for control, 8 for duration 1, 7 for duration 2, 7 for duration 3, and 7 for duration 5. At days 7, 9, and 12 after the laser treatment, the crusts were smaller in the control group and the one-day group than in the other groups (Figure 3A). At days 14 and 21 the scars were difficult to discern at the sites of the control group and the one-day group while the others were evident. The first postnatal hair cycle of mice is about 25–28 days, and the subsequent hair cycle is about 3–5 weeks [22,23]. With the progression from the telogen to anagen phase, the skin color grew darker from day 7 to day 21 after laser treatment. At day 21, the hair cycle was synchronous between the laser-treated site and surrounding skin in the control and one-day groups but asynchronous in the other groups. Microscopically, the epithelial gap was smaller in the control and one-day groups at day 9 and grew larger as the administration duration increased (Figure 3B). At day 9, the crust areas and the epithelial gaps were not significantly different in the control and the one-day groups but they were significantly larger in groups that received betamethasone for two, three, and five days (Figure 3C). Analyzed with one-way ANOVA, crust areas (*p* < 0.0001) and epithelial gaps (*p* < 0.05) were significantly affected by betamethasone in a dosage-dependent manner.

### 3.4. Dosage-Dependent Effect of Topical Corticosteroids Increased Myofibroblast Differentiation and Collagen Deposition

**Figure 4:**
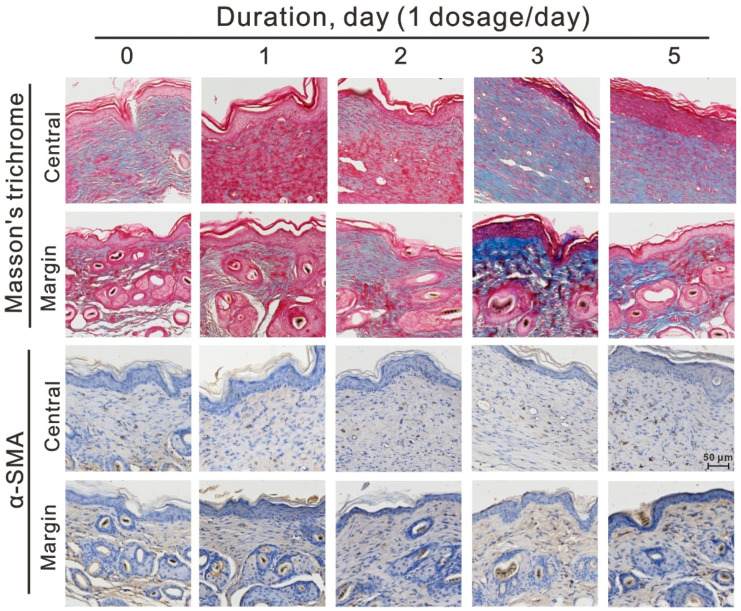
The dosage-dependent effects of topical corticosteroids on myofibroblast differentiation and collagen deposition. Representative photomicrographs of repaired tissue stained with Masson’s trichrome stain (**top**) and α-SMA immunohistochemistry stain (**bottom**) from mice with different administration durations at day 21 after treatment. *n* = 6, scale bar = 50 μm.

**Figure 5:**
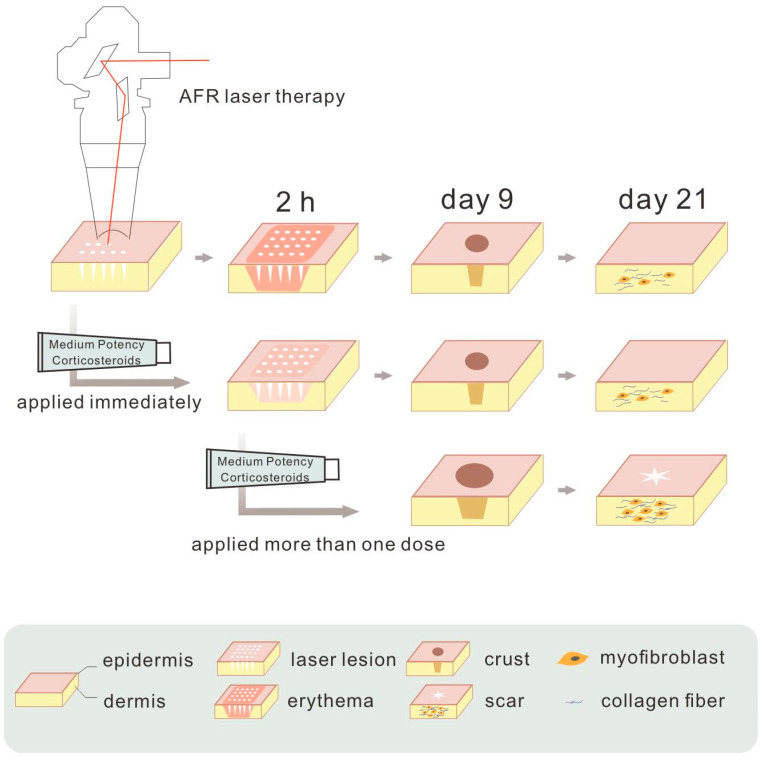
Summary of our findings. One dose of medium potency topical corticosteroid applied immediately after ablative fractional resurfacing (AFR) laser treatment could prevent erythema effectively with minimal disruption to wound healing. When more than one dose was administered, wound healing was delayed, and scar contracture was aggravated.

We examined the dosage effects of topical corticosteroids application on myofibroblast differentiation and collagen deposition. Mice received betamethasone for zero (control group), one, two, three, and five days. Scar tissue was harvested at day 21 after CO_2_ laser treatment and stained with α-SMA immunohistochemistry stain and Masson’s trichrome stain (Figure 4). Myofibroblasts express abundant α-SMA in the dermis layer. In both the center and margins of the scar tissue, there were fewer α-SMA positive cells in the control and one-day groups than in the other groups. Masson’s trichrome stain turns blue to reveal collagen deposition, and microscopic examination of the tissue samples showed fewer collagen deposits in both the center and margins of the scar tissue in the control and one-day groups than in the other groups. These results suggest that applying medium-potency topical corticosteroids to CO_2_ laser-induced wounds for more than one day could increase myofibroblast differentiation and collagen deposition in a dosage-dependent manner (Figure 5).

## 4. Discussion

In this study, we examined the anti-inflammatory and wound healing interference effects of a medium-potency topical corticosteroid on AFR laser-induced wounds. The results indicated the following: (1) topical application was the optimal route of corticosteroid administration for alleviating AFR laser-induced inflammatory responses; (2) in order to decrease the AFR laser-induced erythema, topical corticosteroids are optimally applied immediately after laser treatment; (3) medium-potency topical corticosteroids prolonged healing time, disrupted epithelialization, and increased scar contracture in a dosage-dependent manner; (4) a single dose of medium-potency topical corticosteroids applied immediately after AFR laser treatment effectively prevented erythema with minimal disruption to wound healing.

In a clinical setting, inflammatory response-induced erythema usually develops within a few minutes after laser treatment and gradually resolves within one week. The degree of redness and recovery time are related to the treatment energy [8]. In this study, we found that medium-potency topical corticosteroids applied immediately after laser treatment was the optimal treatment regimen to prevent laser-induced transient erythema. This might be attributable to corticosteroids blocking the inflammatory response prior to erythema development. The inflammatory responses to laser treatment are caused by cutaneous thermal damage which leads to cell necrosis [24] and results in arachidonic acid being released from the cell membrane phospholipids via the catalyzation of the phospholipase A2 [25]. Corticosteroids block phospholipase A2 and depress the release of arachidonic acid from the cell membrane, consequently inhibiting the synthesis of potent inflammatory mediators [15]. Additionally, they inhibit antigen and cytokine production and reduce the proliferation of lymphocytes [12]. These properties all help to diminish post-laser inflammatory reactions such as redness and swelling. Moreover, the vasoconstriction effects of corticosteroids also contribute to a reduction in the redness of laser treated areas [18].

We chose a CO_2_ AFR laser for this study as it is widely used to treat many kinds of deeper skin flaws and frequently causes erythema and small wounds, thus providing a suitable wound model to investigate the anti-inflammatory and wound healing interference effects of topical corticosteroids. Mouse skin is very thin, resulting in thermal injury and capillary coagulation necrosis in the laser-treated areas. Impaired blood supply causes the original wound to expand with dozens of tiny wounds fusing to a single large wound. The total fused wound area was about 50% of the laser treatment area, and the wounds heal 12–14 days after laser treatment. Since the capillary bed is deeper in human skin, wound fusion might not be as serious after clinical CO_2_ AFR laser treatment and wounds made by CO_2_ AFR laser in humans usually heal within 7 days [26]. In this study however, the enlarged wounds in the animal model made it easier to assess from the appearance whether corticosteroids had an effect on wound healing.

Our results showed that medium-potency topical corticosteroids prolonged healing time, disrupted epithelialization, and increased the scar contracture in a dose-dependent manner. Theoretically, corticosteroids may inhibit fibroblast proliferation by inhibiting transforming growth factor-beta and decreasing collagen production [16,27]. However, α-SMA expression and collagen deposition increased when multiple doses of corticosteroids were administered. This may be attributable to the corticosteroids prolonging the wound healing process, which would induce an accumulation of excess collagen and might extend the presence of myofibroblasts, leading to scar contracture [28,29]. Moreover, when multiple doses of corticosteroids were administered, the hair cycles of laser treated sites and surrounding skin were asynchronous. These results indicate that delayed wound healing interfered with the hair cycle and induced scar contracture.

In this study, we found that medium-potency topical corticosteroids could prevent the transient erythema caused by acute inflammation. However, with multiple doses of topical corticosteroids, the wound healing process was delayed in a dosage-dependent manner. Therefore, to prevent transient erythema and avoid delayed wound healing, this study suggests that medium-potency topical corticosteroids be applied as a single dose only, immediately after laser treatment. Clinically, some patients may suffer from prolonged or diffuse erythema after laser therapy, which might be related to residual thermal damage, infection, and allergic or irritant contact dermatitis. Corticosteroids might be considered as a treatment option in such cases but should be applied with caution. A previous study recommended the application of topical corticosteroids after the wound re-epithelialization to reduce the impact of corticosteroids on wound healing and to ensure that their anti-inflammatory effects do not result in increased risk of infection or worsen infection [30].

A variety of drugs are available that offer varying degrees of relief of laser treatment-induced erythema. However, the administration of medium potency topical corticosteroids is far more convenient than that of other drugs. For example, 0.5% piroxicam gel, a NSAID drug, must be covered with an occlusive dressing for 45 min before laser treatment [9,11]; MAS063DP lotion and 0.02% triamcinolone acetonide lotion (a low potency steroid) must be applied twice daily for seven days [10]; and arnebia euchroma ointment must be applied four times a day for one week [31]. Therefore, medium potency topical corticosteroids are preferable to prevent laser treatment-induced erythema.

Laser induced post-inflammatory hyperpigmentation is another major side effect in patients with type II–V skin, however, this particular side effect was not able to be evaluated in the mouse model. Another limitation of this animal model is that mice have thinner skins than humans. Therefore, clinical trials are required in the future. Although the anatomical structure of porcine skin is close to that of human skin, it is too thick for the purposes of this study. The laser settings and the drug diffusion would be vastly different in porcine and human skins. Thus, we chose mice to perform this animal study. This model can also provide a platform to compare the anti-inflammatory efficiency of different corticosteroids or other drugs.

## 5. Conclusions

This study demonstrated that a medium-potency topical corticosteroid could decrease AFR laser-induced inflammatory responses but would disrupt wound healing unless administered as a single dose only. Hence, we suggested that a single dose of medium-potency topical corticosteroid be applied immediately after AFR laser treatment for the prevention of transient erythema and reduction of the side effects of corticosteroids. This intervention might also be effective in suppressing inflammation and with minimal disruption to wound healing.
